# Misalignment between coronavirus financial aid and public health policies: negative incentives for outpatient clinics in the United States

**DOI:** 10.1057/s41271-020-00256-9

**Published:** 2020-09-25

**Authors:** Edward Kim, Kristin Ko

**Affiliations:** 1 Medical School for International Health, Faculty of Health Sciences, Ben Gurion University of the Negev, Be’er Sheva, Israel; 2 Department of Physiology and Biophysics, Georgetown University, Washington, DC USA

**Keywords:** Coronavirus, COVID-19, Policy, SARS-CoV2, Telemedicine

## Abstract

The United States Coronavirus Aid, Relief, and Economic Security Act (CARES Act) led to creation of the Paycheck Protection Program, as well as an expansion of reimbursements for telemedicine. CARES Act drafters over emphasized maintaining employment and overlooked negative downstream effects the policies had on outpatient clinics. The misalignment between this financial aid package and public health policy is most apparent in the pressure administrators face to maintain clinic operations, without a transition plan to adopt telemedicine and associated best practices. If this continues, the result will be suboptimal clinical practices and an increased risk of COVID-19 infection to both staff and patients. Particularly in times of crisis, financial aid packages should not be evaluated in isolation; policymakers should consider their implications for public health while designing, enacting, and implementing such measures.

## Introduction

The United States (US) Congress enacted the Coronavirus Aid the United States on 27 March 2020 [1]. This law authorized distribution of $349 billion in relief aid to American workers and small businesses, and the expansion of reimbursement for telemedicine visits [2]. It also enabled creation of four temporary loan programs, managed by the Small Business Administration (SBA), namely the Paycheck Protection Program (PPP), Economic Injury Disaster Loan and Emergency Advance, SBA Express Bridge Loans, and SBA Debt Relief [3].

Many individuals and small businesses experienced significant financial hardship as a result of public health measures to minimize the spread of coronavirus disease 2019 (COVID-19), including social distancing and stay-at-home orders, [1]. In just 1 month, the number of unemployed increased from 1.4 to 7.1 million [4]. The CARES Act is an attempt to offset the economic impact of the federal, state, and local public health activities to limit COVID-19 transmission. As of 15 April 2020, the PPP program had disbursed $342 billion USD through 1.6 million loans—with allocation of 40 billion, or almost 12% of available funding to healthcare and social assistance organizations [5].

While the CARES Act helps to mitigate the impact of unprecedented levels of unemployment and to sustain struggling businesses, it has also changed the financial incentive structure of outpatient clinics. These incentives may induce poor practice decisions—such as staffing and operating outpatient clinics without strict adherence to public health guidelines—and delay recovery efforts by increasing risk of infection from COVID-19.

## Paycheck Protection Program

The Paycheck Protection Program (PPP) is a program of loans distributed by the US Small Business Administration (SBA) in conjunction with the Department of the Treasury; it expands the existing 7(a) loan program. It helps businesses to retain their employees by providing money to cover payroll costs—including vacation, medical and sick leave—along with rent, mortgage interest, and utilities [6]. Most importantly, the government will forgive the loan if 75% of the borrowed amount is allocated to payroll costs, and the number of employees and their levels of compensation are maintained [1].

## Financial factors influence staffing decisions in outpatient clinics

Similarly, after an outpatient clinic is approved for a loan, the drafters of the law expected the practice to maintain its staff and their usual pay. Because the loan does not specify contingencies for adhering to public health guidelines, it incentivizes each practice to continue operations with its full, pre-COVID-19 loan staff. In many states, a clinic is considered an ‘essential business’ that means clinics can continue operating despite stay-at-home orders [7]. The loans cover costs incurred from 15 February 2020 through 30 June 2020, a period during which there was an expectation of many new cases of COVID-19 throughout the United States [1]. Although many governing bodies have prescribed general guidelines on how to operate businesses during the COVID-19 pandemic, their lack of enforcement allows outpatient clinics to selectively follow some and disregard others with impunity. This could result in a fully staffed office that does not abide by all public health guidelines [8–12].

To minimize the financial impact of the economic downturn and the cost of retaining staff, many clinical practices will continue operating during these times with inadequate resources and loose adherence to the guidelines [13]. There are two opposing forces at work, namely: (1) for paid employees to go to their workplaces to continue to generate revenue for the practice and (2) reduce working hours for the sake of safe practices.

## Telemedicine changes the use of clinical support staff

There has also been a major push for clinicians to adopt telemedicine practices, as an effective substitute for traditional face-to-face visits in times of a pandemic [14]. As of 6 March 2020 under the CARES Act and the 1135 waiver, the Centers for Medicare and Medicaid Services (CMS) expanded their telehealth service and reimbursement policies [15]. Although the recent reimbursement expansion promotes adoption of telemedicine at a national level, the widespread lack of experience using the platform will likely lead to poor rates of adoption and inadequate practices [16]. The gap between the anticipated benefits and actual effectiveness of telemedicine has undermined efforts to transition [17]. Given these considerations, the program incentives clinical administrators to shift their approach from availability–to utilization of their staff.

Substantial up-front planning is required to understand the existing issues and reduce the downstream complexity involved in restructuring workflow [18]. Beyond learning the technological infrastructure, there are barriers associated with disrupted workflow. Changes to traditional roles of clinical support staff can be subsumed under two categories: undefined and changed roles, and alignment with clinical processes [17]. “Undefined and changed roles” is best understood as a change in responsibility for a particular task or the assignment of a new one after adopting telemedicine. “Alignment with clinical processes” refers to barriers confronted when telemedicine does not integrate into nor support existing practices.

Practicing through telemedicine also entails a range of new liabilities, such as licensure, billing, privacy, and confidentiality concerns [19, 20]. Without adequate preparation, telemedicine can force practices to rely on ‘workarounds’ to overcome systemic shortcomings, increasing the likelihood of poor outcomes [21]. For instance, introducing new workflow will increase practice owners’ perceived liabilities and may encourage them to maintain current workflow that prevents staff from working remotely. Additionally, not all diseases can be adequately addressed in a remote setting [22]. Even practices that have already adopted telemedicine into their workflow may find the current model unfeasible during an infectious disease outbreak-even subspecialty physicians that serve rural locations work with nurses who can visit their patients to perform physical examinations [23]. These factors contribute to the pressure to maintain staff in an office setting, potentially relying on suboptimal practices, and thus increasing risk of infection to both staff and patients [24].

## Discussion

The misalignment between this financial aid package and public health policy is most apparent in the pressure administrators' face to maintain their pre-COVID-19 outpatient practice norms, without a transition plan to adopt telemedicine and associated best practices. This disconnect between fiscal and public health strategies can result in inefficient interventions and limit improving health outcomes within the United States [25].

Changing financial incentives can foster behavioral change even in medical settings [26]. Hence, poor planning and program design by policymakers create the risk of producing poor outcomes and undermine progress by imposing greater disease burden on patients who are at the highest risk. Considering that the US Congress enacted the PPP program and the government implemented it rapidly—“14 years’ worth of loans in less than 14 days”—there was insufficient time to involve all stakeholders in the core discussions and the roll-out strategy [27].

In retrospect, the CARES Act, specifically the PPP, could have better addressed the needs of outpatient practices by including two minor provisions that would ensure employee safety and promote the adoption of telemedicine without unnecessarily complicating the program. As it stands, the policy forgives the borrowed amount if a business strictly adheres to employment requirements.

- *Safe working conditions* Policymakers should have also required businesses, particularly health organizations, to provide safe working conditions for employees. That is, businesses should be required, where defined by national, state, and local policies, to stipulate that reasonable accommodations be made for employees to work from home if safety measures cannot be met. Similar to the current policy, any failure to follow such requirements could lead to loan repayment.
- *Expand ‘rent’ to include telehealth investment* In the PPP’s current form, a portion of the borrowed amount can be dedicated to covering the cost of rent. The rent category could be expanded for medical practices to include investments in telehealth infrastructure and services. Just as a clinic’s walls provide a setting for the provision of health services, the infrastructure for telehealth allows clinicians to provide comparable, while safe, services throughout the pandemic.

Considering the PPP was set to expire at the end of June 2020, it is critical to identify important gaps and limitations to improve future iterations. Overall, the PPP is limited in the support and improvement it offers the healthcare system insofar as it addresses employment as both a process and an outcome. *Future policymakers should consider employment as an outcome objective only.* Financial assistance directed towards telemedicine and associated infrastructure should instead be considered as a process objective. If funding were dedicated to providing telemedicine, it would allow an office to continue practicing and generating revenue. This would incentivize administrators to adopt a new delivery model better suited to the challenges of COVID-19, and employees could redefine their roles while continuing to provide value—all while pushing patient care into a safer setting. This alternative fiscal program aligns the needs and values of all stakeholders—the general public, patients, clinical administrators, and their staff.

## Conclusion

The current lack of enforcement, informal guidelines, and the fact that outpatient clinics are eligible for loan forgiveness from the PPP program (but lack prior experience using telemedicine), means that many clinics will continue to be fully staffed, resulting in an increased risk of exposure to coronavirus for clinicians and patients alike. This will likely prolong the spread of the virus and make recovery more difficult. While rapid distribution of financial resources is critical during an economic downturn, it should not come at the expense of misaligned policies that create incentives that undermine public health.

The financial impact of COVID-19 is substantial, not only within the United States but worldwide. As other countries develop similar programs to address economic downturn, policymakers should consider the shortcomings of the CARES Act noted here. Doing so would enable future financial programs to better support healthcare organizations and patients, particularly in an outpatient setting, and avoid the negative downstream effects of poor program design.
